# Supplementary material for: Coupled protein synthesis and ribosome-guided piRNA processing on mRNAs
Source: Nat Commun. 2021 Oct 13;12:5970. doi: 10.1038/s41467-021-26233-8 (PMC8514520; doi:10.1038/s41467-021-26233-8)
Supplement: Supplementary file 1 — Supplementary Figures and Tables [file 41467_2021_26233_MOESM1_ESM.pdf]

Supplementary Information

Coupled protein synthesis and ribosome-guided piRNA processing on mRNAs

Yu H. Sun, Ruoqiao Huiyi Wang, Khai Du, Jiang Zhu, Jihong Zheng, Li Huitong Xie, Amanda A. Pereira, Chao Zhang, Emiliano P. Ricci, and Xin Zhiguo Li

Correspondence:

Xin_Li@URMC.rochester.edu (X.Z.L.)

This PDF file includes:

Supplementary Figures 1-7

Supplementary Table 1

Supplementary Figures

Supplementary Fig. 1

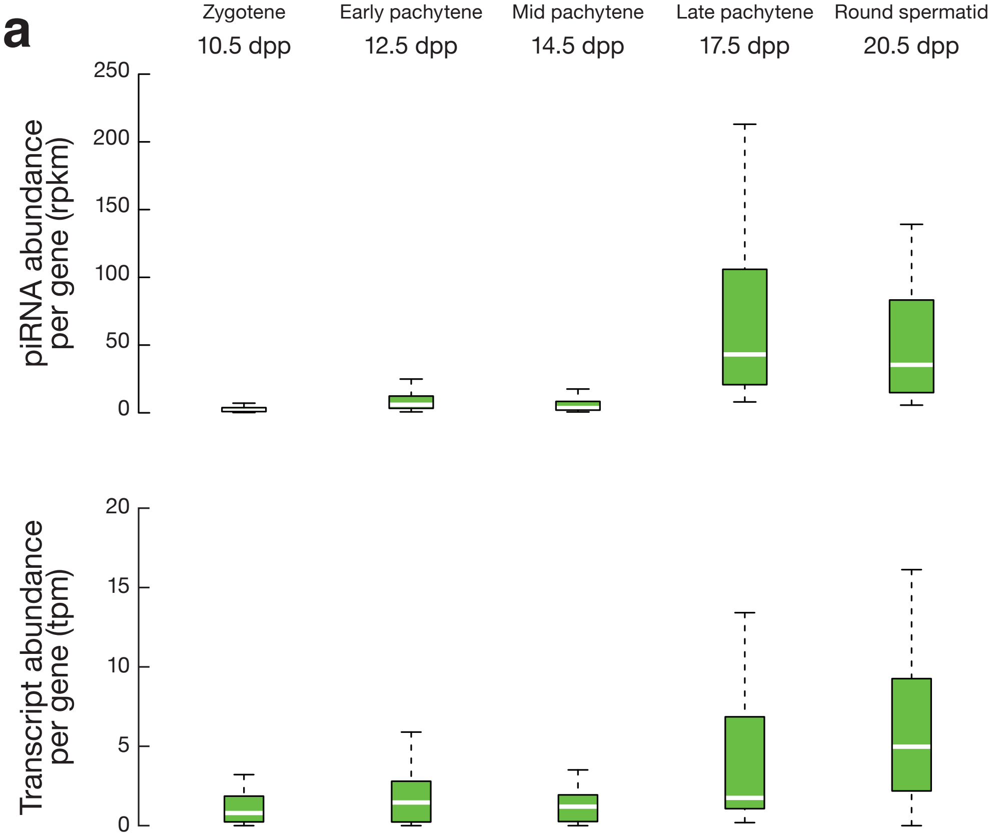

Supplementary Fig. 1

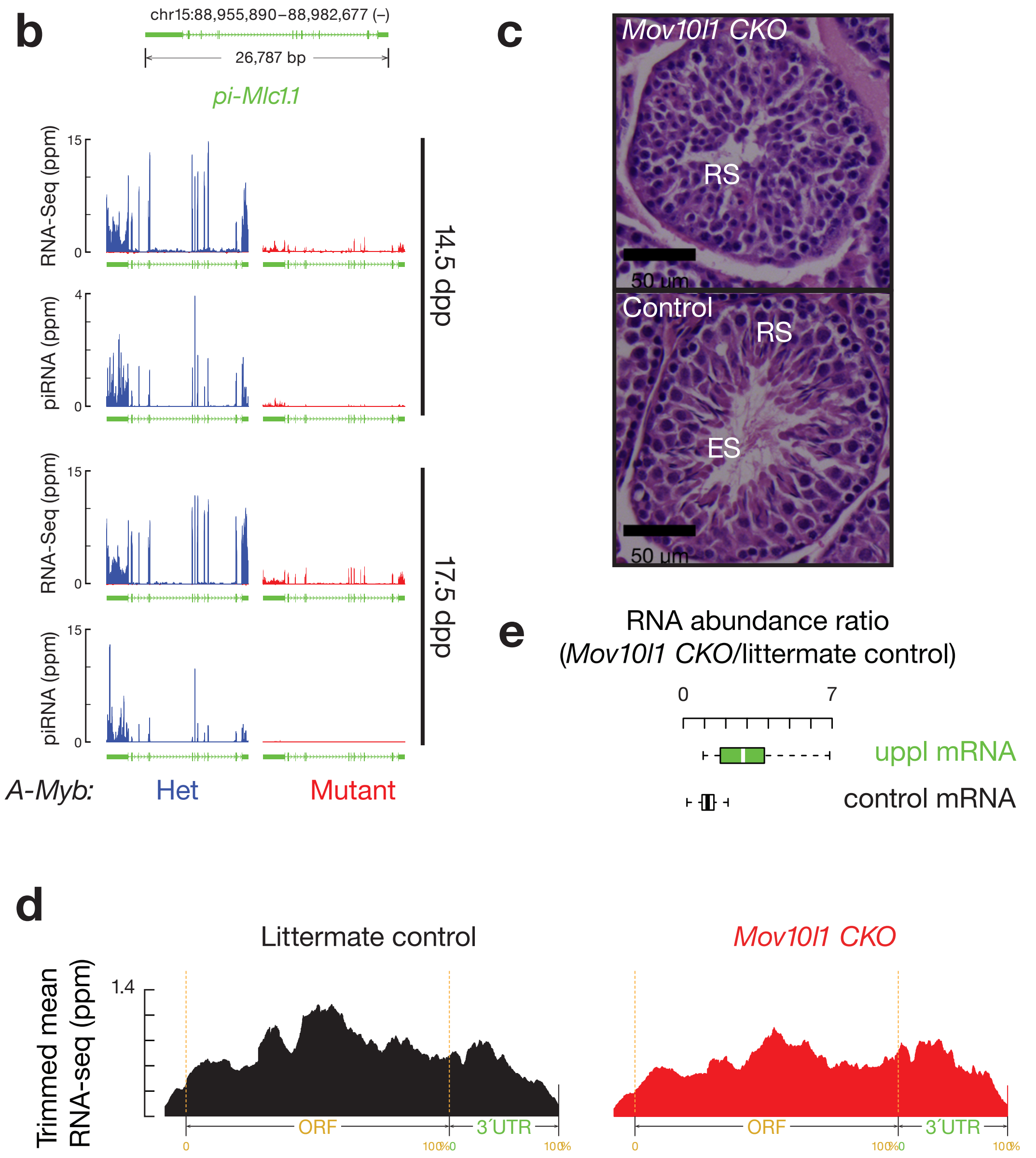

Supplementary Fig. 1

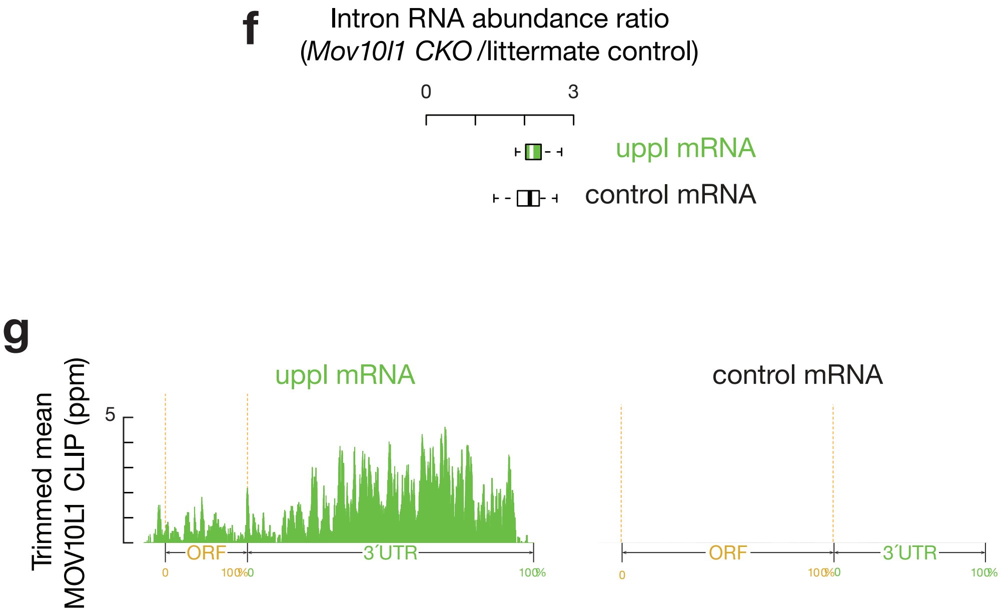

Supplementary Fig. 1, Related to Fig. 1. A-MYB regulates 3´UTR piRNA precursor transcription and MOV10L1 regulates their processing. (**a**) Top, boxplots represent 3´UTR piRNA density per gene as spermatogenesis progresses in reads per kilobase per million mapped reads (rpkm). Bottom, boxplots represent uppl transcript expression, as measured by RNA-seq in transcripts per million (tpm), at each of the five wild-type development stages examined. (**b**) Transcript and piRNA abundance in heterozygous (Het) and homozygous *A-Myb* (Mut) point mutant testes are shown for an illustrative example of uppl mRNA at 14.5 and 17.5 dpp: the 3´UTR piRNA gene *pi-Mlc1.1*. Ppm, parts per million reads mapped to the genome. (**c**) Histology of adult testis sections from *Mov10l1*$^{CKO/\Delta}$ *Neurog3-cre* (*top*) and *Mov10l1*$^{CKO/\Delta}$ (*bottom*). RS, round spermatids; ES, elongating spermatids. Experiments were repeated at least three times independently with similar results. Scale bar, 50 μm. (**d**) Aggregated data for RNA abundance from *Mov10l1*$^{CKO/\Delta}$ testes (*left*) and *Mov10l1*$^{CKO/\Delta}$ *Neurog3-cre* (*right*) across 5´UTRs, ORFs, and 3´-UTRs of the control mRNAs from adult testes. The x-axis shows the median length of these regions, and the y-axis represents the 10% trimmed mean of relative abundance. Ppm, parts per million. (**e**) Boxplots of RNA abundance ratios of *Mov10l1*$^{CKO/\Delta}$ *Neurog3-cre* versus *Mov10l1*$^{CKO/\Delta}$ adult testis (here and elsewhere, uppl mRNAs in green and control mRNAs in black). Control mRNA n=43, uppl mRNA n=30. (**f**) Boxplots of intron RNA abundance ratios of *Mov10l1*$^{CKO/\Delta}$ *Neurog3-cre* versus *Mov10l1*$^{CKO/\Delta}$ adult testis. Control mRNA n=43, uppl mRNA n=30. (**g**) Aggregated data for MOV10L1 CLIP-seq abundance (10% trimmed mean) on uppl mRNAs (left) and control mRNAs (right) from wild-type testis[13]. Box plots in (**a**), (**e**) and (**f**) show the 25th and 75th percentiles, whiskers represent the 5th and 95th percentiles and midlines show median values.

Supplementary Fig. 2

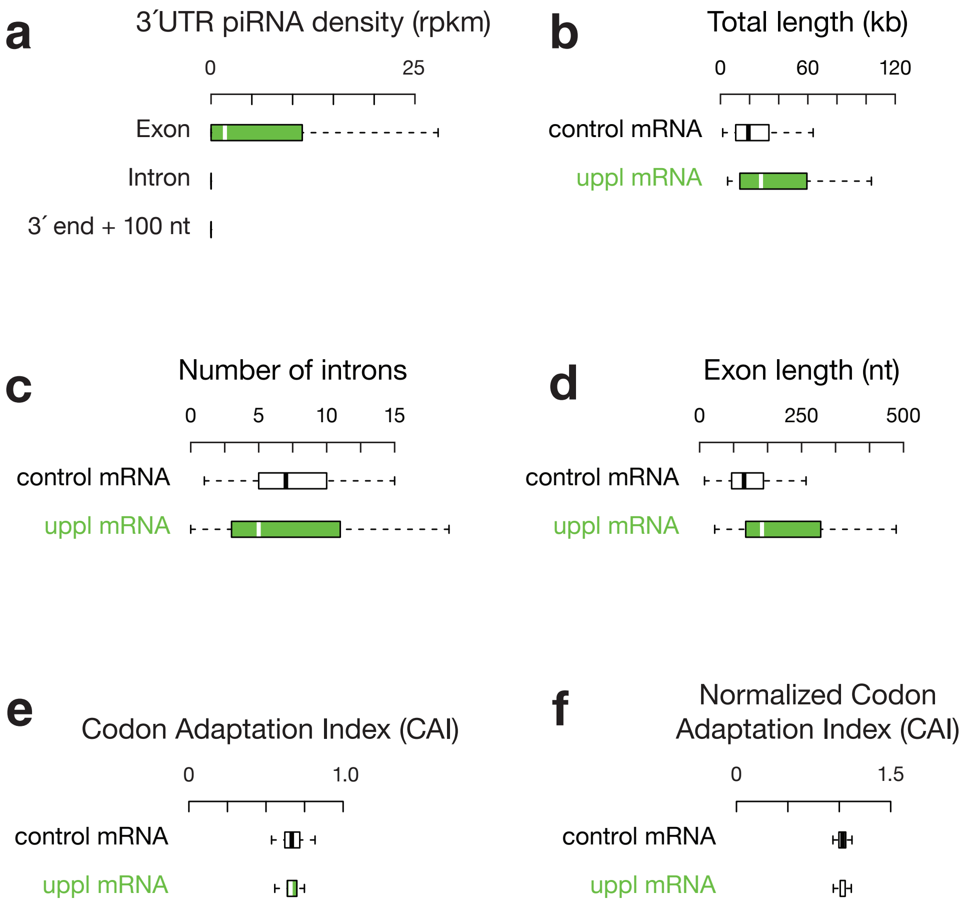

Supplementary Fig. 2, Related to Fig. 2. uppl mRNAs are bifunctional, coding for proteins and piRNAs. (**a**) Boxplots showing the density of uniquely mapping piRNAs in the exons, introns, and 100 nt after the 3´end (i.e., the polyadenylation site, PAS) of uppl mRNAs. uppl mRNA n=30. (**b**) Boxplots showing unspliced transcript length distributions. Control mRNA n=43, uppl mRNA n=30. (**c**) Boxplots showing number of introns. Control mRNA n=43, uppl mRNA n=30. (**d**) Boxplots showing exon length distributions. Control mRNA n=43, uppl mRNA n=30. (**e**) Boxplots showing codon adaptation index (CAI)[69,70]. Control mRNA n=43, uppl mRNA n=30. (**f**) Boxplots showing normalized codon adaptation index (CAI)[152]. Control mRNA n=43, uppl mRNA n=30. Box plots in (**a**) to (**f**) show the 25th and 75th percentiles, whiskers represent the 5th and 95th percentiles and midlines show median values.

Supplementary Fig. 3

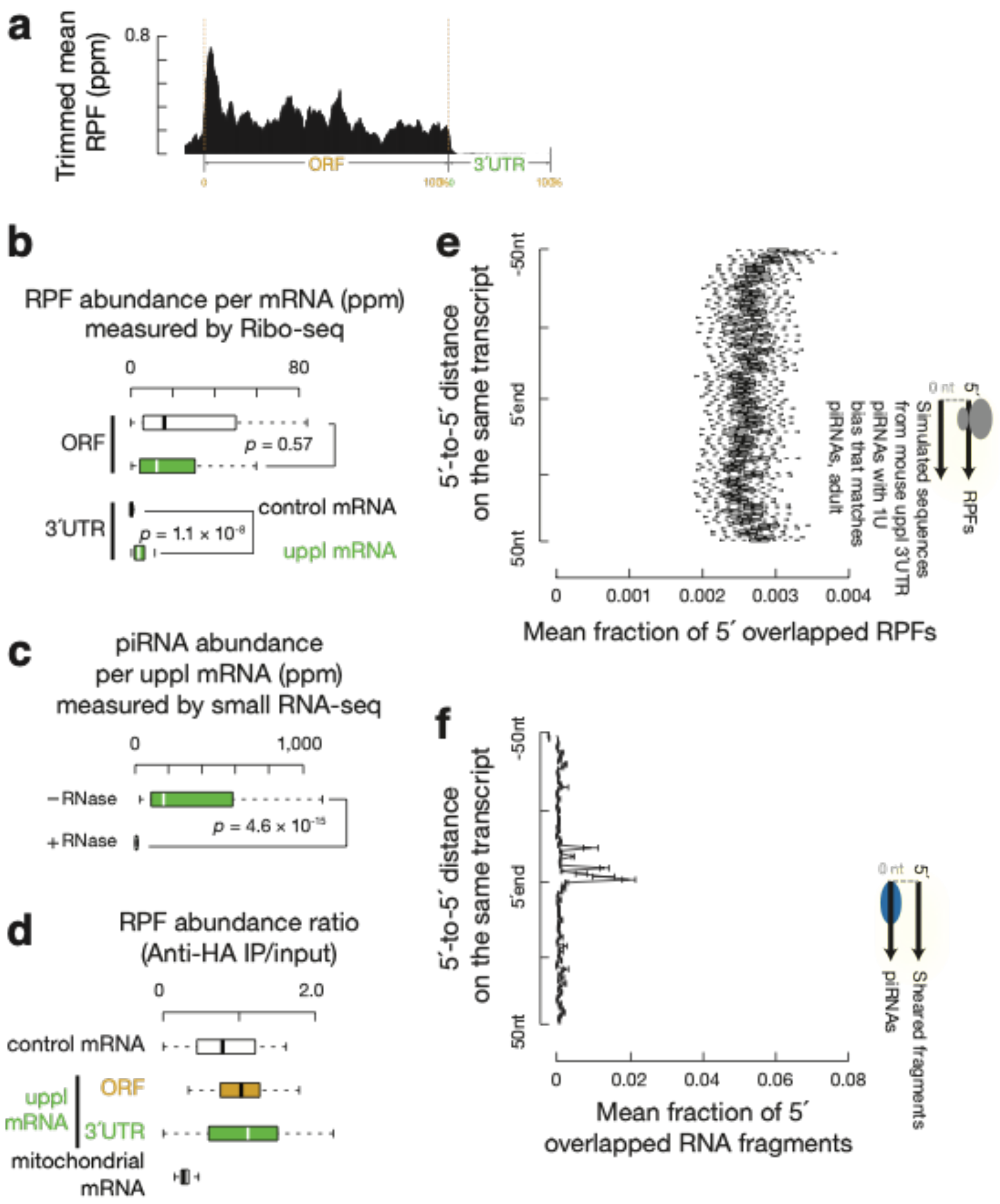

Supplementary Fig. 3, Related to Fig. 3. Ribosomes serve as templates for endonucleolytic cleavage to form 3´UTR piRNA 5´ends. (**a**) Metagene plots of RPFs at 5´UTR, ORF, and 3´UTR of the control mRNAs in adult testes. The x-axis shows the median length of these regions, and the y-axis represents the mean of normalized abundance. Ppm, parts per million. (**b**) Boxplots of the RPF abundance per mRNA at ORF and 3´UTR in adult testes. p value was determined by paired two-sided Wilcoxon rank sum test. Control mRNA

n=43, uppl mRNA n=30. (**c**) Boxplots of 3´UTR piRNA abundance per uppl mRNA with and without RNase A & T1 treatment in adult wild-type testes. p value was determined by paired two-sided Wilcoxon rank sum test. uppl mRNA n=30. (**d**) Boxplots of RFP abundance in anti-HA IP versus input (before IP) (*n* = 3, n is the number of biological replicates of deep sequencing data used to generate the figures.). The Ribo-seq libraries before (input) and after anti-HA IP were normalized to reads mapping to mRNA ORFs in adult RiboTag testes. (**e**) Boxplots of distance spectra of 5´-ends of RPFs from adult testis that overlap simulated sequences. The 5´-end overlap analyses between RPFs and simulated sequences were computed n=10,000 times. uppl mRNA n=30. (**f**) Boxplots of distance spectra of 5´-ends of sheared RNA fragments that overlap piRNAs of adult testes. uppl mRNA n=30. Data are presented as mean values +/- SD. Box plots in (**b**), (**c**), (**d**) and (**e**) show the 25th and 75th percentiles, whiskers represent the 5th and 95th percentiles and midlines show median values.

Supplementary Fig. 4

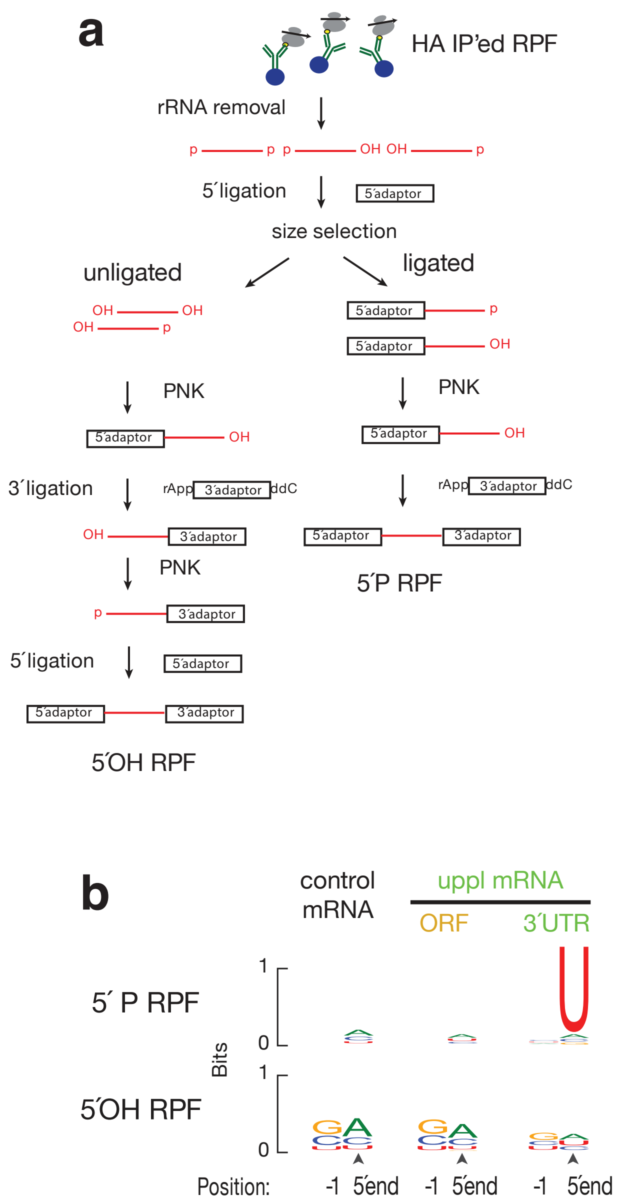

Supplementary Fig. 4

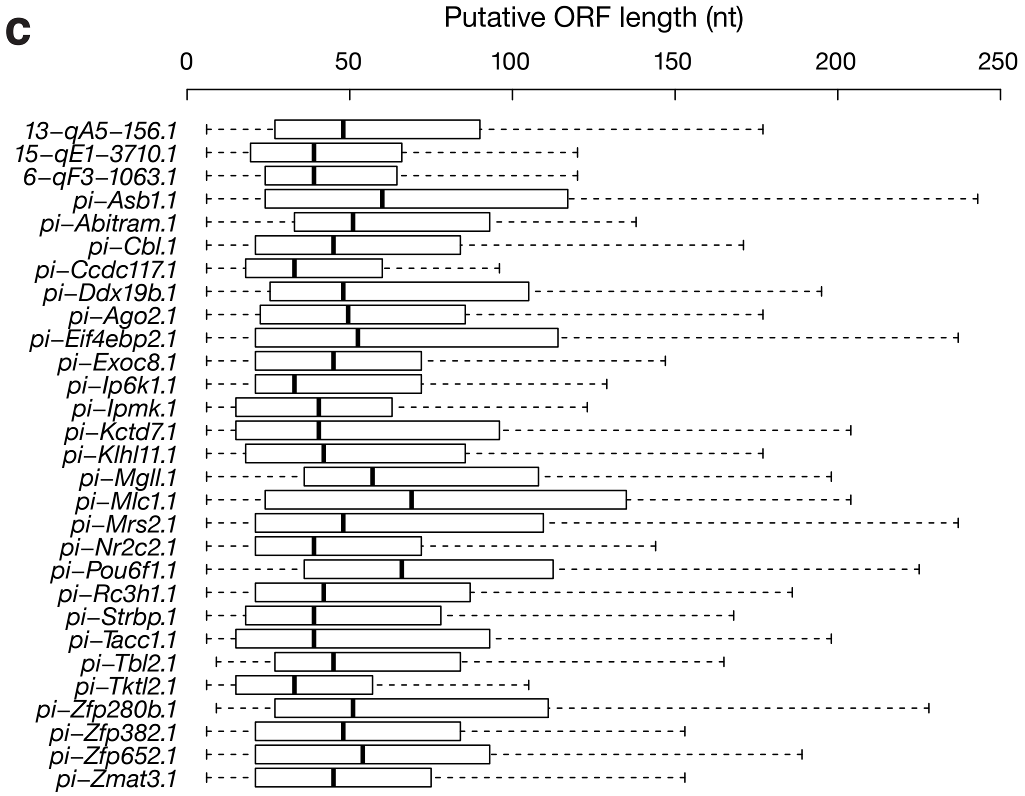

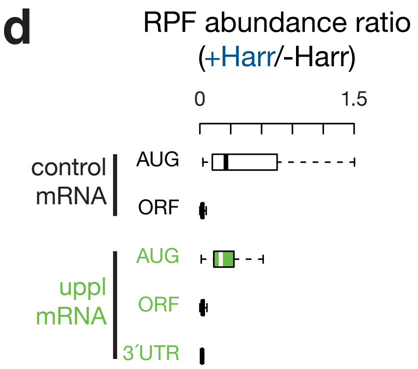

Supplementary Fig. 4

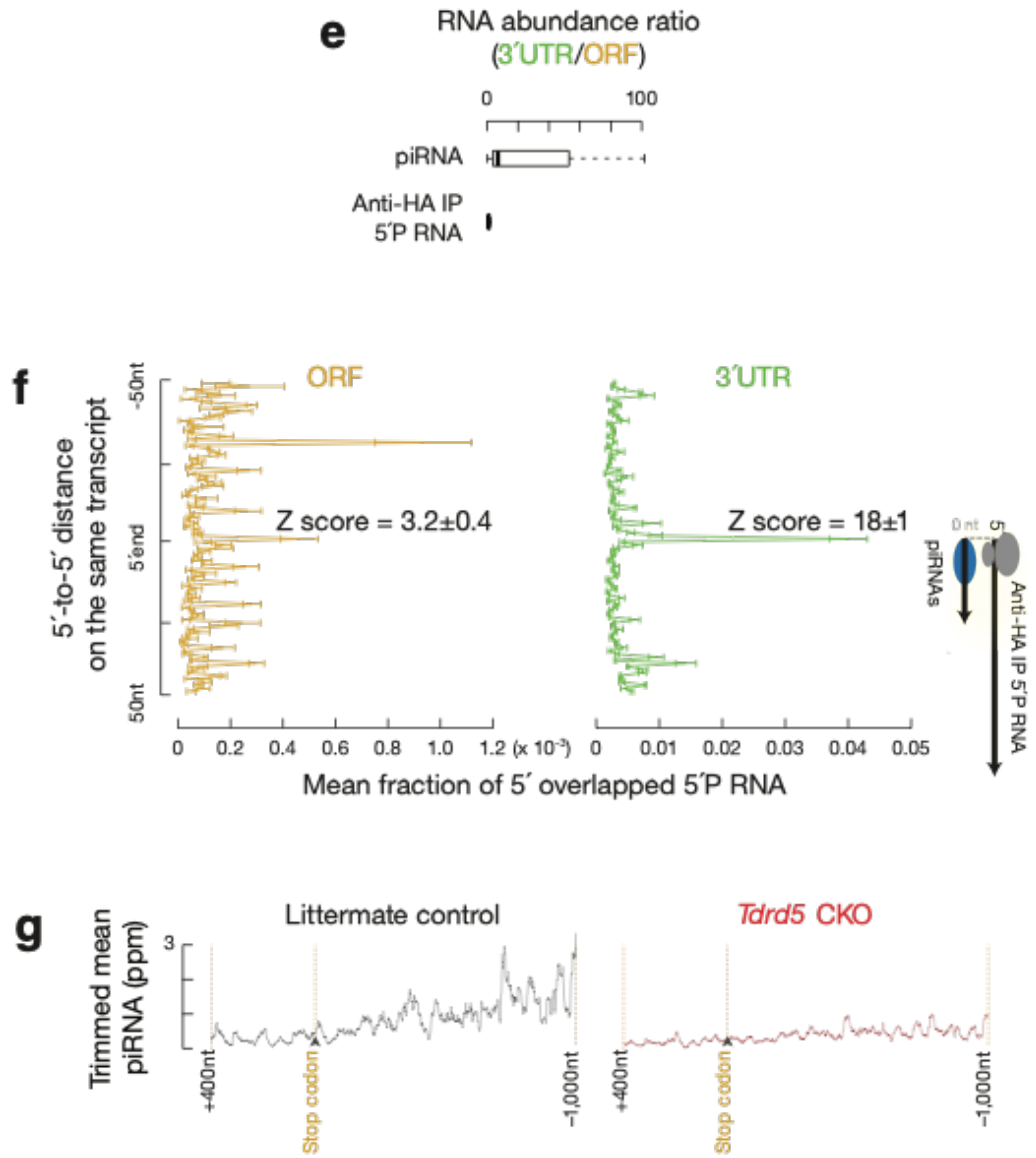

Supplementary Fig. 4

h

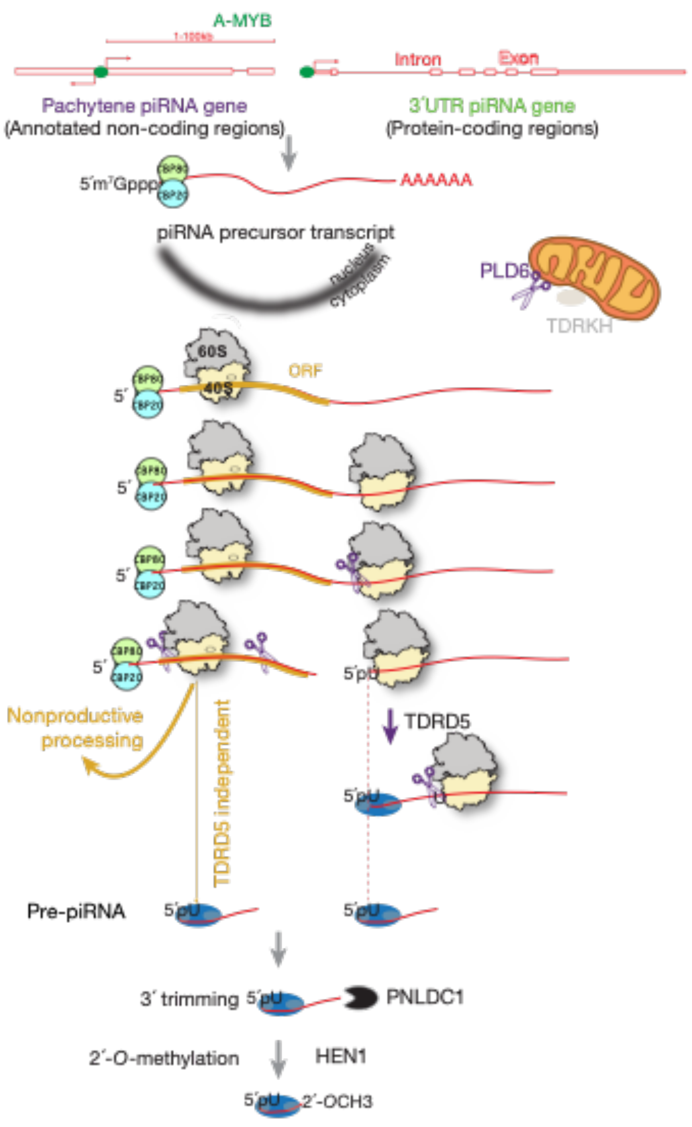

Supplementary Fig. 4, Related to Fig. 4. Biphasic piRNA biogenesis at ORFs and 3´UTRs. (**a**) Schematic of the modified ribosome profiling library construction procedure which captures 5´OH RPF (*left*) and 5´P RPFs (*right*). (**b**) Sequence logos depicting nucleotide bias at 5´-ends and 1 nt upstream of 5´-ends. Top to bottom: anti-HA immunoprecipitated RPF species with a 5´monophosphate end, and anti-HA immunoprecipitated RPF species with a 5´OH end, from adult testes. (**c**) Boxplots of the length of all putative ORFs embedded in each uppl 3´ UTR. uppl mRNA n=30. n for each uppl mRNA varies. (**d**) Boxplots of change in RPF abundance with harringtonine treatment (*n*=2 independent biological replicates) versus no treatment adult wild-type testes. Control mRNA n=43, uppl mRNA n=30. (**e**) Boxplots of the ratios of RNA

abundance (normalized with length) at 3´UTRs versus RNA abundance at ORFs of uppl mRNAs in adult wild-type testes. uppl mRNA n=30. (**f**) Distance spectrum of 5´-ends of anti-HA immunoprecipitated 5´P RNA species from ORFs (*left*) and from 3´UTRs (*right*) that overlap piRNAs in adult testes (*n*=3 independent biological replicates). Data are mean ± standard deviation. (**g**) Metagene analysis of piRNA abundance from 400 nt upstream stop codon to 1,000 nt downstream stop codon from adult wild-type testes. Ppm, parts per million. (**h**) A general model of ribosome-guided piRNA biogenesis. The grey and tan bubbles represent the large and small subunits of ribosomes respectively. The blue bubbles represent PIWI proteins. Grey circle in PIWI proteins on the left represents MID domain that recognizes the 5´-phosphate (5´P), on the right represents PAZ domain, in between represents PIWI domain. piRNA precursors are synthesized by RNA polymerase II, and contain the 5´-cap, exons, introns, and a poly(A) tail. The transcription of pachytene piRNA genes and 3´UTR piRNA gene is controlled by A-MYB. The PLD6-mediated cleavage before uridine generates strings of head-to-tail phased piRNAs that will be further trimmed and methylated. Ribosomes translate the piRNA precursors in a canonical fashion through initiating at the start codon near the 5´-cap. Post-termination ribosomes traverse in the 3´ direction. The 5´-end-loaded PIWI protein and the downstream ribosome specify the endonuclease PLD6 cleavage site, and determine the 5´- and 3´-ends of head-to-tail strings before 3´-end trimming. piRNA biogenesis at the 3´ UTRs requires TDRD5 protein. Transcripts containing ORFs are processed to piRNAs inefficiently that does not require TDRD5 (Gold arrows). This model applies to precursor source of both lncRNAs and mRNAs. Box plots in (**c**), (**d**) and (**e**) show the 25th and 75th percentiles, whiskers represent the 5th and 95th percentiles and midlines show median values.

Supplementary Fig. 5

a

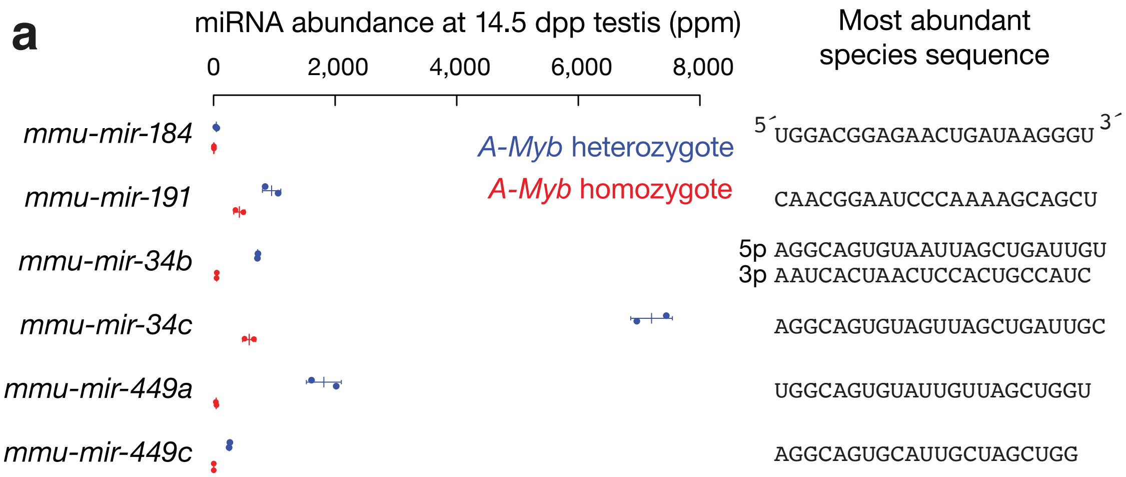

Supplementary Fig. 5

b

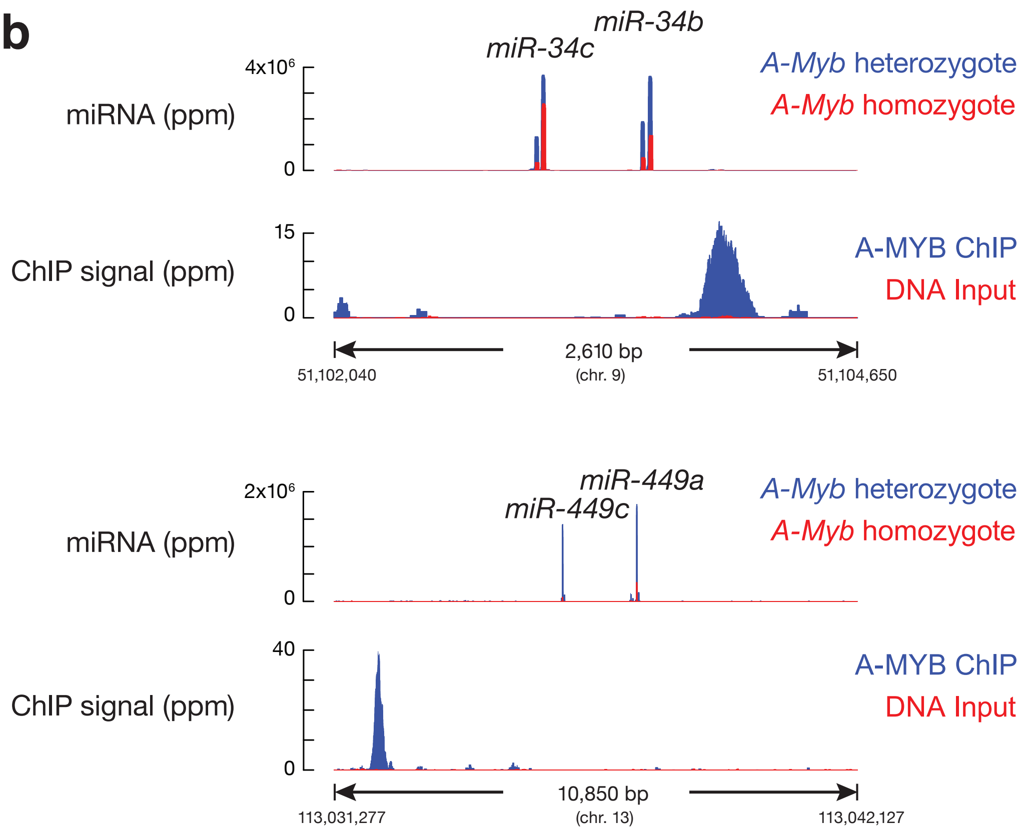

c

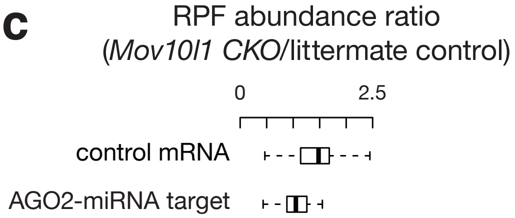

Supplementary Fig. 5, Related to Fig. 5. Co-translational piRNA processing. (**a**) miRNA abundance in *A-Myb* mutant and heterozygous testes at 14.5 dpp. (sample size *n* = 2 independent biological samples). Data are mean ± standard deviation. (**b**) A-MYB ChIP-seq and input signal near the two miRNA genes that are decreased in *A-Myb* mutant. For each gene, the figure also reports the miRNA abundance in *A-Myb* mutant and heterozygous testes at 17.5 dpp. (**c**) Boxplots of the changes of RPF abundance in *Mov10l1* CKO mutants (*n*=3 independent biological replicates) compared to littermate controls in adult testes. Box plots show the 25th and 75th percentiles, whiskers represent the 5th and 95th percentiles and midlines show median values.

Supplementary Fig. 6

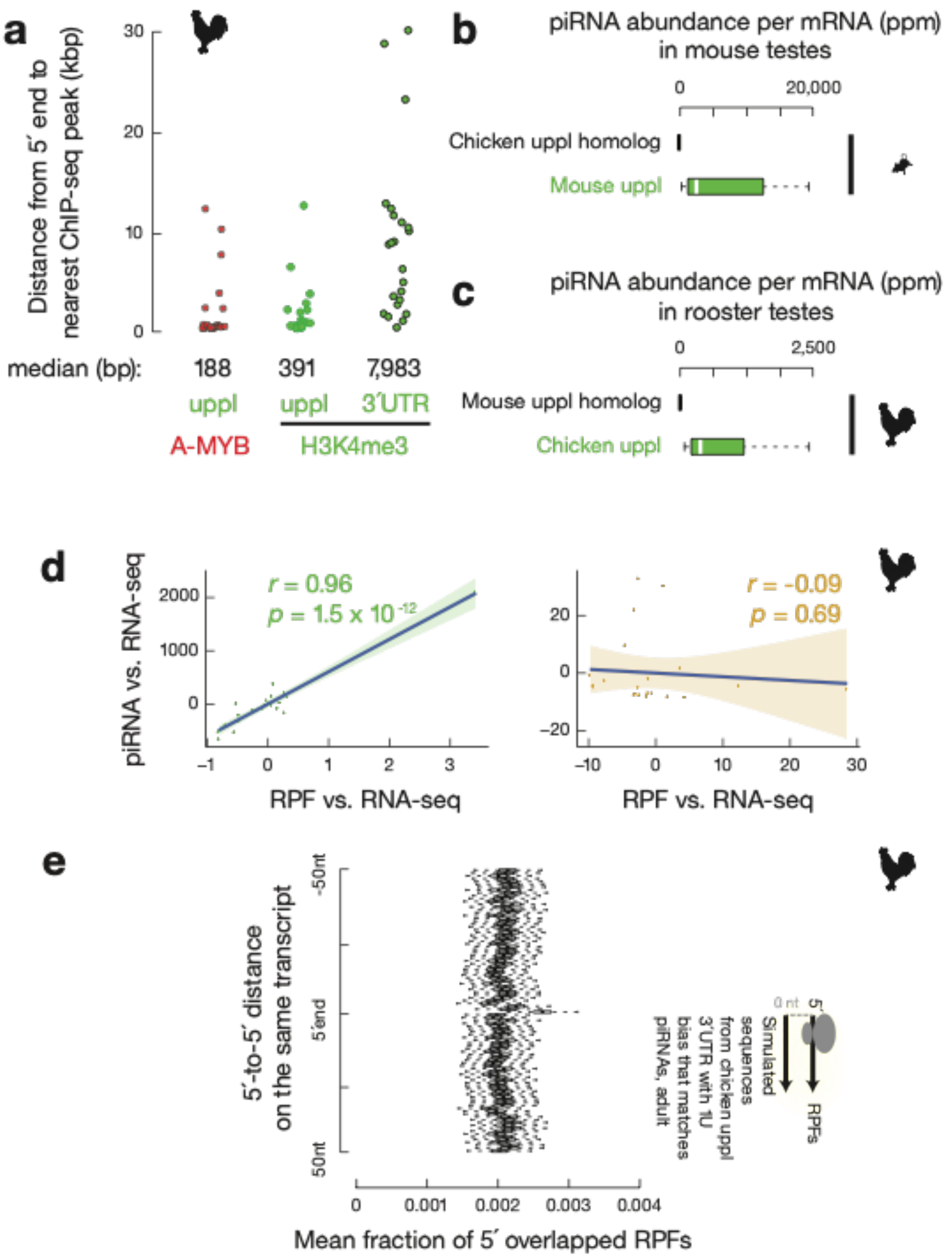

Supplementary Fig. 6, Related to Fig. 6. Conserved piRNA biogenesis from mRNA 3´UTRs. (**a**) The distance from the annotated transcription start site of each uppl mRNA gene (middle) and from the 5´-ends of uppl mRNA 3´UTRs (right) to the nearest H3K4me3 peak, and

from the annotated transcription start site of each uppl mRNA gene to the nearest A-MYB peak (left) in roosters. (**b**) Boxplots of piRNA abundance per mRNA in adult wild-type mouse testes. Control mRNA n=43, uppl mRNA n=30. (**c**) Boxplots of piRNA abundance per mRNA in adult rooster testes. Control mRNA n=23, uppl mRNA n=23. (**d**) Residual plot of partial correlation between piRNA and RPF abundance conditional on RNA-seq abundance at the chicken uppl 3´UTR (left) and uppl ORF (right) in adult rooster testes. Linear regression was performed between piRNA and RNA-seq abundance, RPF and RNA-seq abundance on uppl mRNA 3´UTRs separately, and residuals were plotted with smooth parameter 'method=lm'. Data are presented as mean values +/- SD shown as error bands. (**e**) Boxplots of distance spectra of 5´-ends of RPFs from adult rooster testis that overlap simulated sequences. The 5´-end overlap analyses between RPFs and simulated sequences were computed n=10,000 times. Chicken uppl mRNA n=23. Box plots in (**b**), (**c**) and (**e**) show the 25th and 75th percentiles, whiskers represent the 5th and 95th percentiles and midlines show median values.

Supplementary Fig. 7

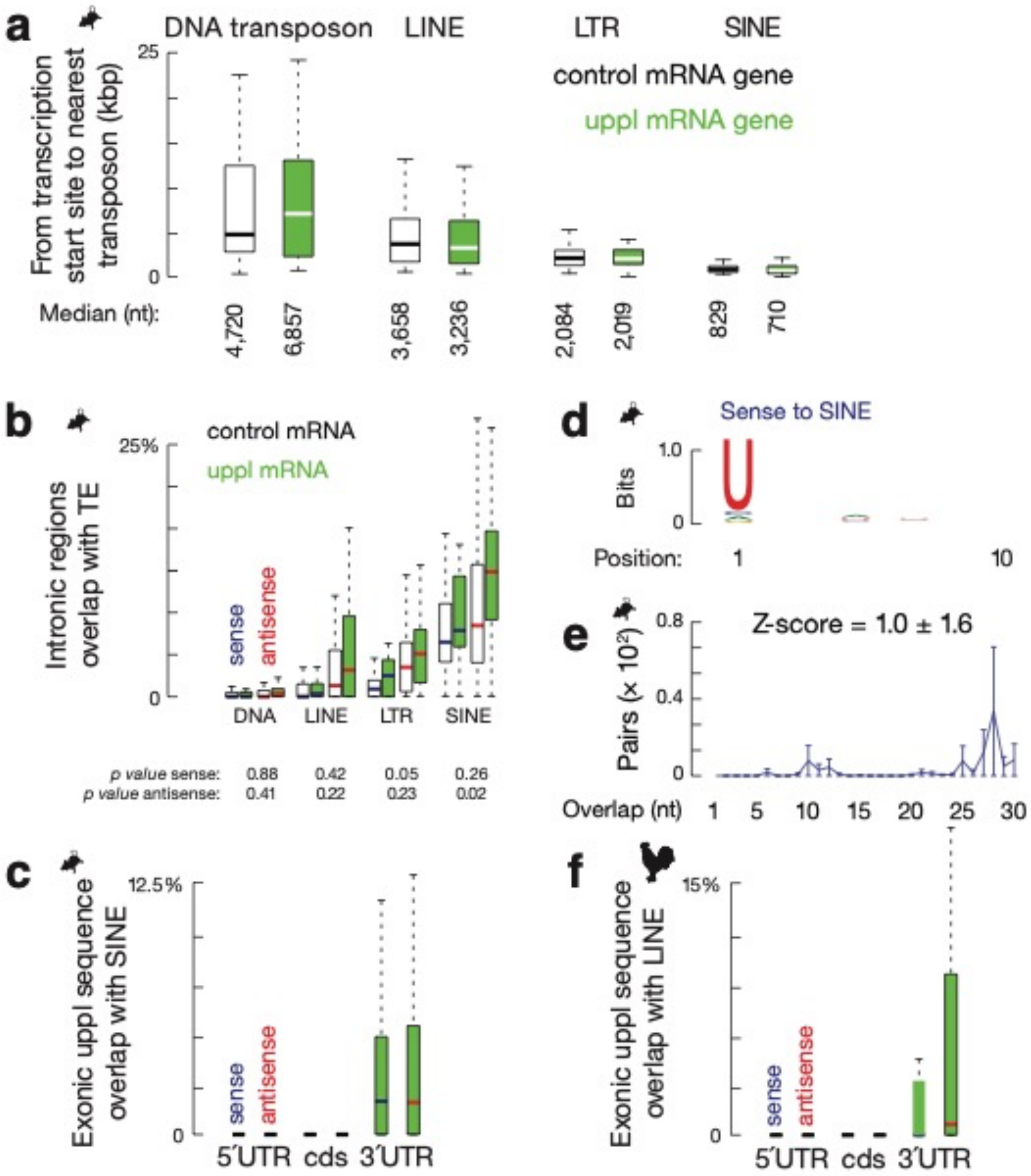

Supplementary Fig. 7, Related to Fig. 7. piRNA precursor mRNAs contain transposon fragments. (**a**) The distance from the uppl mRNA gene and control mRNA gene to the nearest annotated TE superfamilies in mouse genome. Control mRNA n=43, uppl mRNA n=30. (**b**) Boxplots showing the fraction of transcript intron sequence correspondence to sense (blue) and anti-sense (red) transposon sequences in mouse genome. Control mRNA n=43, uppl mRNA n=30. (**c**) Boxplots showing the fraction of transcript exon sequence correspondence to sense (blue) and antisense (red) SINE sequences in mouse genome. uppl mRNA n=30. (**d**) Sequence logo showing the nucleotide composition of sense SINE-piRNA species that uniquely map to

mouse uppl mRNAs. (**e**) The 5′-5′ overlap between piRNAs from opposite strands was analyzed to determine if sense SINE piRNAs from mouse uppl mRNAs display Ping-Pong amplification in trans. The number of pairs of piRNA reads at each position is reported. The *Z*-score indicates that a significant ten-nucleotide overlap (“Ping-Pong”) was detected. *Z*-score = 1.96 corresponds to *p*-value = 0.025. Data are presented as mean values +/- SD from n=3 biological replicates. (**f**) Boxplots showing the fraction of transcript exon sequence correspondence to sense (blue) and antisense (red) LINE sequences in chicken genome. Chicken uppl mRNA n=23. Box plots in (**a**), (**b**), (**c**) and (**f**) show the 25th and 75th percentiles, whiskers represent the 5th and 95th percentiles and midlines show median values.

Supplementary Tables

Supplementary Table 1

Name	Sequence
Degenerate primer	5´-GCACCCGAGAATTCCANNNNNNNNC-3´
Spike-in RNA	5´-UUACAUAAGAUAUGAACGGAGCCCmG -3´
Small RNAseq and Ribo-seq 3´ adapter	5′-TGGAATTCTCGGGTGCCAAGG-3´

Supplementary Table 1, Primer, Spike-in RNA and Adapter
