# Supplementary material for: Coupled protein synthesis and ribosome-guided piRNA processing on mRNAs
Source: Nat Commun. 2021 Oct 13;12:5970. doi: 10.1038/s41467-021-26233-8 (PMC8514520; doi:10.1038/s41467-021-26233-8)
Supplement: Supplementary file 4 — Description of additional supplementary files [file 41467_2021_26233_MOESM4_ESM.docx]

Description of additional supplementary information

Title: Supplementary Data 1.

Description: Detailed information and statistics for the sequencing data used in this study (species refers to numbers of unique sequences in a set of reads).

(A) Ribo-seq statistics: reads and species

(B) Small RNA sequencing statistics: reads and species.

(C) RNA-seq statistics: reads and species.

(D) Degradome-seq statistics: reads and species.

(E) Genome coordinates for the 30 3´UTR piRNA precursors with annotated ORFs provided in UCSC BED format (i.e., 0-based) for mm10.

(F) Genome coordinates for the 43 control mRNAs with annotated ORFs provided in UCSC BED format (i.e., 0-based) for mm10.

(G) Genome coordinates for the 23 piRNA precursor mRNAs with annotated ORFs provided in UCSC BED format (i.e., 0-based) for galgal6.

(H) Genome coordinates for the 23 uppl chicken homolog mRNAs with annotated ORFs provided in UCSC BED format (i.e., 0-based) for galgal6.
