# Supplementary material for: Coupled protein synthesis and ribosome-guided piRNA processing on mRNAs
Source: Nat Commun. 2021 Oct 13;12:5970. doi: 10.1038/s41467-021-26233-8 (PMC8514520; doi:10.1038/s41467-021-26233-8)
Supplement: Supplementary file 5 — Reporting Summary [file 41467_2021_26233_MOESM5_ESM.pdf]

nature portfolio

Corresponding author(s): Xin Zhiguo Li

Last updated by author(s): Sep 12, 2021

Reporting Summary

Nature Portfolio wishes to improve the reproducibility of the work that we publish. This form provides structure for consistency and transparency in reporting. For further information on Nature Portfolio policies, see our Editorial Policies and the Editorial Policy Checklist.

Statistics

For all statistical analyses, confirm that the following items are present in the figure legend, table legend, main text, or Methods section.

n/a	Confirmed	
☐	☒	The exact sample size (*n*) for each experimental group/condition, given as a discrete number and unit of measurement
☐	☒	A statement on whether measurements were taken from distinct samples or whether the same sample was measured repeatedly
☐	☒	The statistical test(s) used AND whether they are one- or two-sided<br>*Only common tests should be described solely by name; describe more complex techniques in the Methods section.*
☒	☐	A description of all covariates tested
☐	☒	A description of any assumptions or corrections, such as tests of normality and adjustment for multiple comparisons
☐	☒	A full description of the statistical parameters including central tendency (e.g. means) or other basic estimates (e.g. regression coefficient) AND variation (e.g. standard deviation) or associated estimates of uncertainty (e.g. confidence intervals)
☐	☒	For null hypothesis testing, the test statistic (e.g. *F*, *t*, *r*) with confidence intervals, effect sizes, degrees of freedom and *P* value noted<br>*Give P values as exact values whenever suitable.*
☒	☐	For Bayesian analysis, information on the choice of priors and Markov chain Monte Carlo settings
☒	☐	For hierarchical and complex designs, identification of the appropriate level for tests and full reporting of outcomes
☒	☐	Estimates of effect sizes (e.g. Cohen's *d*, Pearson's *r*), indicating how they were calculated

Our web collection on statistics for biologists contains articles on many of the points above.

Software and code

Policy information about availability of computer code

Data collection	RNAseq data were generated on HiSeq 2000 instrument (Illumina, San Diego, CA, USA). Histology and immunostaining images were taken using Leica DM4000 B LED microscope system with Leica software: Leica Application Suite X (v1.1.0.12420). Western Blotting images were taken using Azure c300 imaging system, with cSeries capture software (v1.6).
Data analysis	General analyses of RNA sequencing, small RNA sequencing, Ribo-seq and degradome sequencing data were performed using piPipes pipeline (v1.4): https://github.com/bowhan/piPipes. A Ribo-seq pipeline was developed for this study: https://github.com/LiLabZhaohua/RiboSeqPipeline. The script for calculating spectral distribution from simulated negative controls: https://github.com/LiLabZhaohua/LiLabScripts/blob/master/spectral_mid_100.py. For RNA-seq reads, the expression per transcript was normalized to the top quartile of expressed transcripts per library calculated by Cufflinks (v2.2.1), and the tpm (transcripts per million) value was quantified using Salmon (v0.8.2). ChIP peaks were identified using MACS2 (v2.1.1.20160309). Degradome reads were aligned to the genome using TopHat (v2.0.12). 5´-end overlap analysis was performed using our own script (https://gist.github.com/nimezhu/d8734d2ae6c1619218f1). Chicken transcriptome was assembled using StringTie (v1.3.3b) and transcriptome annotation was perform using TransDecoder (v5.5.0), BlastP (v2.10.0+) and Hmmer (v3.3). Statistical analyses were performed in R (v3.5.0). Nucleotide periodicity was computed using GeneCycle (v1.1.4) package in R. Ribosome release score (RRS) was computed using ORFik (v1.6.0) package from Bioconductor (Release 3.10). qPCR data were analyzed using DART-PCR (v1.0). For mouse transcriptome annotation, 30 uppl mRNAs defined in our previous studies with mm9 were converted to mm10 coordinates with using using liftOver website: https://genome.ucsc.edu/cgi-bin/hgLiftOver. Mass Spectrometry data were analyzed using SEQUEST within the Proteome Discoverer software platform, v2.2 (Thermo Fisher) employing the SwissProt mouse database. Codon Adaptation Index was calculated using: DAMBE v7.2.1 and CAIcal v1.4. miRNA target search was performed by miRanda (v3.3).

For manuscripts utilizing custom algorithms or software that are central to the research but not yet described in published literature, software must be made available to editors and reviewers. We strongly encourage code deposition in a community repository (e.g. GitHub). See the Nature Portfolio guidelines for submitting code & software for further information.

Data

Policy information about availability of data

All manuscripts must include a data availability statement. This statement should provide the following information, where applicable:

- Accession codes, unique identifiers, or web links for publicly available datasets
- A description of any restrictions on data availability
- For clinical datasets or third party data, please ensure that the statement adheres to our policy

Next-generation sequencing data used in this study have been deposited at the NCBI Gene Expression Omnibus under the accession number GSE155350 (https://www.ncbi.nlm.nih.gov/geo/query/acc.cgi?acc=GSE155350). Mass spectrometry data have been uploaded to the ProteomeXchange Consortium via the PRIDE database under accession number PXD027489.

Field-specific reporting

Please select the one below that is the best fit for your research. If you are not sure, read the appropriate sections before making your selection.

☒ Life sciences ☐ Behavioural & social sciences ☐ Ecological, evolutionary & environmental sciences

For a reference copy of the document with all sections, see nature.com/documents/nr-reporting-summary-flat.pdf

Life sciences study design

All studies must disclose on these points even when the disclosure is negative.

Sample size	We use mice in congenic background to minimize the individual variation. Our prior experience demonstrates that three replicates provide superb statistical power in sequencing analysis, allowing the elimination of false positive and negative changes in gene, RPF or piRNA expression.
Data exclusions	no data were excluded for analysis.
Replication	We include at least 3 biological replicates to compute our p value. All replicates were successful.
Randomization	NA
Blinding	Blinding is not relevant to our study as we include both positive and negative controls in each step of experiment procedure.

Reporting for specific materials, systems and methods

We require information from authors about some types of materials, experimental systems and methods used in many studies. Here, indicate whether each material, system or method listed is relevant to your study. If you are not sure if a list item applies to your research, read the appropriate section before selecting a response.

Materials & experimental systems

n/a	Involved in the study	
☐	☒	Antibodies
☒	☐	Eukaryotic cell lines
☒	☐	Palaeontology and archaeology
☐	☒	Animals and other organisms
☒	☐	Human research participants
☒	☐	Clinical data
☒	☐	Dual use research of concern

Methods

n/a	Involved in the study	
☒	☐	ChIP-seq
☒	☐	Flow cytometry
☒	☐	MRI-based neuroimaging

Antibodies

Antibodies used	anti-AGO2 mouse monoclonal antibody (Wako Pure Chemical Corporation, 018-22021, Clone Name: 2D4, 1:500 dilution)<br>anti-TUBULIN rabbit antibody (Bimake, Houston, TX, USA, A5105, 1:1000 dilution)<br>sheep anti-mouse IgG-HRP (GE Healthcare, Little Chalfont, UK; NA931V, 1:5,000 dilution)<br>donkey anti-rabbit IgG-HRP (GE Healthcare, NA934V, 1:5,000 dilution)<br>anti-HA mouse antibody (ascites fluid, Covance, Princeton, NJ, USA; MMS-101P, 1:2000 dilution)<br>anti-PELOTA antibody (Thermo, PA5-31697, 1:50 dilution)<br>anti-CBP80 rabbit antibody (Bethyl laboratories, A301-794A, for immunoprecipitation, 1: 50 dilution)<br>anti-eIF4E rabbit antibody (Bethyl laboratories, A301-153A, for immunoprecipitation, 1: 50 dilution)

	Alexa Fluor 488 conjugated secondary antibodies (Molecular Probes, Eugene, OR, USA, secondary antibody, 1:500 dilution)
Validation	anti-AGO2 mouse monoclonal antibody (Wako Pure Chemical Corporation, 018-22021): validated by multiple studies including Molecular cell (2018): 69.2, 265-278.<br>anti-TUBULIN rabbit antibody (Bimake, Houston, TX, USA, A5105): validated by the manufacturer<br>sheep anti-mouse IgG-HRP (GE Healthcare, Little Chalfont, UK; NA931V): validated by the manufacturer and Nature Cell Biology (2021): 22.2, 200-212.<br>donkey anti-rabbit IgG-HRP (GE Healthcare, NA934V): validated by the manufacturer and Nature Cell Biology (2021): 22.2, 200-212.<br>anti-HA mouse antibody (ascites fluid, Covance, Princeton, NJ, USA; MMS-101P): validated in multiple studies including Nature 449.7163 (2007): 731.<br>anti-PELOTA antibody (Thermo, PA5-31697): validated by the manufacturer<br>anti-CBP80 rabbit antibody (Bethyl laboratories, A301-794A): validated by multiple studies including Nature structural & molecular biology (2013): 20.6, 710-717.<br>anti-eIF4E rabbit antibody (Bethyl laboratories, A301-153A): validated by the manufacturer<br>Alexa Fluor 488 conjugated secondary antibodies (Molecular Probes, Eugene, OR, USA): validated by the manufacturer

Animals and other organisms

Policy information about studies involving animals; ARRIVE guidelines recommended for reporting animal research

Laboratory animals	Mice were maintained and used according to guidelines for animal care of the NIH and the University Committee on Animal Resources at the University of Rochester. Mice of the following strains C57BL/6J (Jackson Labs, Bar Harbor, ME, USA; stock number 664); Rpl22tm1.1Psam on a C57BL/6J background (Jackson Labs; stock number 011029)136; Mov10l1tm1.1Jw on a mixed 129X1/SvJ × C57BL/6J background11; and Tg(Neurog3-cre)C1Able/J on a B6.FVB(Cg) background (Jackson Labs; stock number 006333)137 were genotyped as described. Comparisons of compound mutants and controls involving Mov10l1 CKO mutation were performed using siblings from individual litters. Mice were housed in a temperature (64-79°F) and humidity (30-70%) controlled room with 12 light/12 dark cycle. Male mice were collected at certain ages including 20.5dpp, 8 weeks (adult) stage. White Leghorn testes of the Cornell Special C strain from one-year old roosters were used according to guidelines for animal care of the NIH and the University Committee on Animal Resources at the University of Rochester. Chicken samples were collected at the adult stage.
Wild animals	The study did not involve wild animals.
Field-collected samples	The study did not involve sample collected from the wild.
Ethics oversight	Animals were maintained and used according to guidelines for animal care of the NIH and the University Committee on Animal Resources at the University of Rochester.

Note that full information on the approval of the study protocol must also be provided in the manuscript.
